# S1Q3T3 Electrocardiographic Pattern in a Case of Colonic Ileus: A Case Report

**DOI:** 10.7759/cureus.79985

**Published:** 2025-03-03

**Authors:** Ripjeet S Nat, Saif M Srouji, Pal Satyajit Singh Athwal, Mohammed G Elhassan

**Affiliations:** 1 Internal Medicine, Saint Agnes Medical Center, Fresno, USA

**Keywords:** cardiology, cta, ecg, gastroenterology, internal medicine, intestinal ileus, pulmonary embolism, s1q3t3

## Abstract

The S1Q3T3 electrocardiographic (ECG) pattern is commonly described in patients with acute pulmonary embolism (PE). However, it is a nonspecific, nonsensitive ECG finding associated with right heart strain that can be found in other acute presentations as well. Most of these acute diseases are of cardiac or pulmonary origin and pathophysiology. Acute abdominal pathologies are not well-known etiologies for this characteristic ECG pattern. We present a case of postoperative colonic dilation and ileus that showed up as acute, new atrial fibrillation (AF) associated with the S1Q3T3 pattern and that resolved after the resolution of the ileus symptoms.

## Introduction

The S1Q3T3 electrocardiographic (ECG) pattern (also known as the McGinn-White sign) is a characteristic ECG pattern defined as a deep (>1.5 mm) S wave in lead I and deep Q wave and T-inversion in lead III [1]. In the literature, it is most commonly related to pulmonary embolism (PE) [2-5], and in one report, it was one of the clues to the diagnosis even when the D-dimer level was normal, which is a rare occurrence in PE [6]. These electrical patterns often associated with cases of right ventricle (RV) strain usually occur in response to increased RV pressures and/or the dilation of the right ventricle and were found to be linked to the risk of worse outcomes in these patients including hemodynamic instability, circulatory shock, and death [7-10].

While not a specific or sensitive sign for PE, when taken into account with the general clinical picture, it usually increases the suspicion of the presence of PE, thus leading to earlier imaging and diagnosis [11-13]. It was also described in other cases associated with right ventricular (RV) strain and other cardiopulmonary diseases such as pneumothorax, acute bronchospasm, and acute lung diseases causing acute cor pulmonale. To the best of our knowledge, this pattern has not been described in patients with colonic dilation or ileus.

We report a case of new-onset atrial fibrillation (AF) after back surgery associated with the S1Q3T3 pattern that was initially thought to be due to PE but later discovered to be associated with colonic ileus, which is hypothesized in this report to have caused transient obstruction of the inferior vena cava (IVC), thus resulting in decreased venous return to the RV and ensuing RV strain, which resolved after the resolution of abdominal symptoms. This case report aims to describe the occurrence of this pattern in a different pathological process than is usually encountered and to serve as precedence for possible future reports, thus helping the clinician broaden their differential diagnosis when encountering it.

## Case presentation

**Figure 1:**
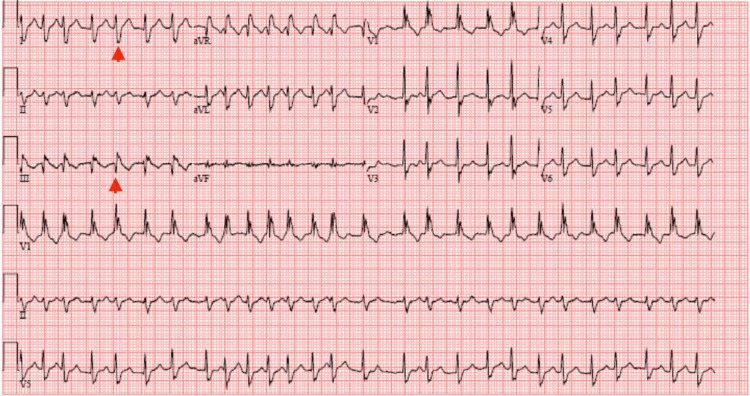
Initial ECG Initial ECG showing atrial fibrillation with rapid ventricular response, dominant R wave in V1, and the S1Q3T3 pattern (red arrowheads) ECG: electrocardiogram

A 65-year-old man with a history of hypertension and hyperlipidemia presented to the emergency department (ED) as a transfer from another hospital after being found to have AF with a rapid ventricular response where multiple attempts of rate and rhythm control were unsuccessful despite the use of intravenous (IV) atrioventricular (AV) blocking agents adenosine and metoprolol tartrate. The patient had undergone a lumbar spinal fusion two days prior and was working with physical therapy when he started complaining of palpitations. He also noted some abdominal discomfort described as bloating and loose stools but denied vomiting or constipation. An ECG was performed at that hospital that showed AF but also demonstrated the S1Q3T3 pattern (Figure 1). He was transferred to our hospital after failure to control his heart rate with intravenous (IV) atrioventricular (AV) blocking agents.

**Figure 2:**
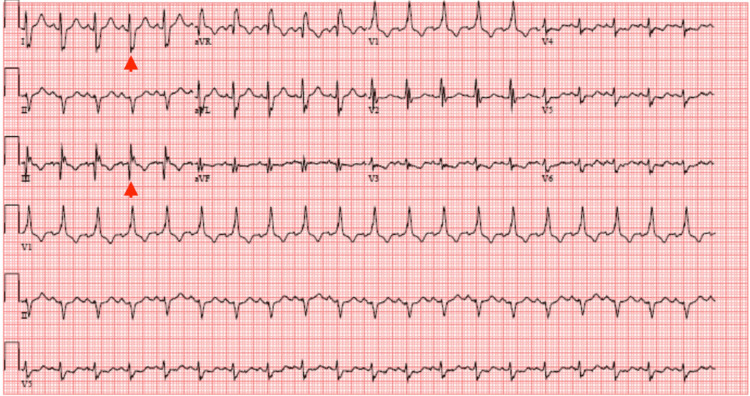
Interim ECG ECG showing conversion to sinus tachycardia and persistent S1Q3T3 pattern (red arrowheads) ECG: electrocardiogram

Upon transportation to the ED, his vital signs were as follows: heart rate of 198 beats per minute (bpm) (irregularly irregular), blood pressure of 121/79 mmHg, respiratory rate of 18 breaths per minute, temperature of 36.6 degree Celsius, and oxygen saturation of 88% on ambient air. Later, he self-reverted to sinus tachycardia with a heart rate of 105 bpm, was started afterward on oral metoprolol succinate 100 mg daily for the maintenance of rate control, and did not require cardioversion. However, despite this, he continued to experience hypoxia and required oxygen supplementation by nasal cannula to maintain adequate blood oxygen saturation. His ECG at this point was showing sinus tachycardia with the persistence of the S1Q3T3 pattern (Figure 2).

Initial laboratory tests revealed only mild hyponatremia at 130 mml/L; mild elevation of aspartate aminotransferase (AST)/alanine aminotransferase (ALT) at 63 and 64 U/L, respectively; mild lactic acidosis at 2.5 mmol/L (reference range: 0.5-2 mmol/L); leukocytosis at 15.4 K/mcL (which were thought to be at the time reactive considering recent surgical intervention); mild elevation of D-dimer at 532 ng/mL (reference range: ≤234 ng/mL); and normal cardiac troponin and thyroid-stimulating hormone levels.

**Figure 3:**
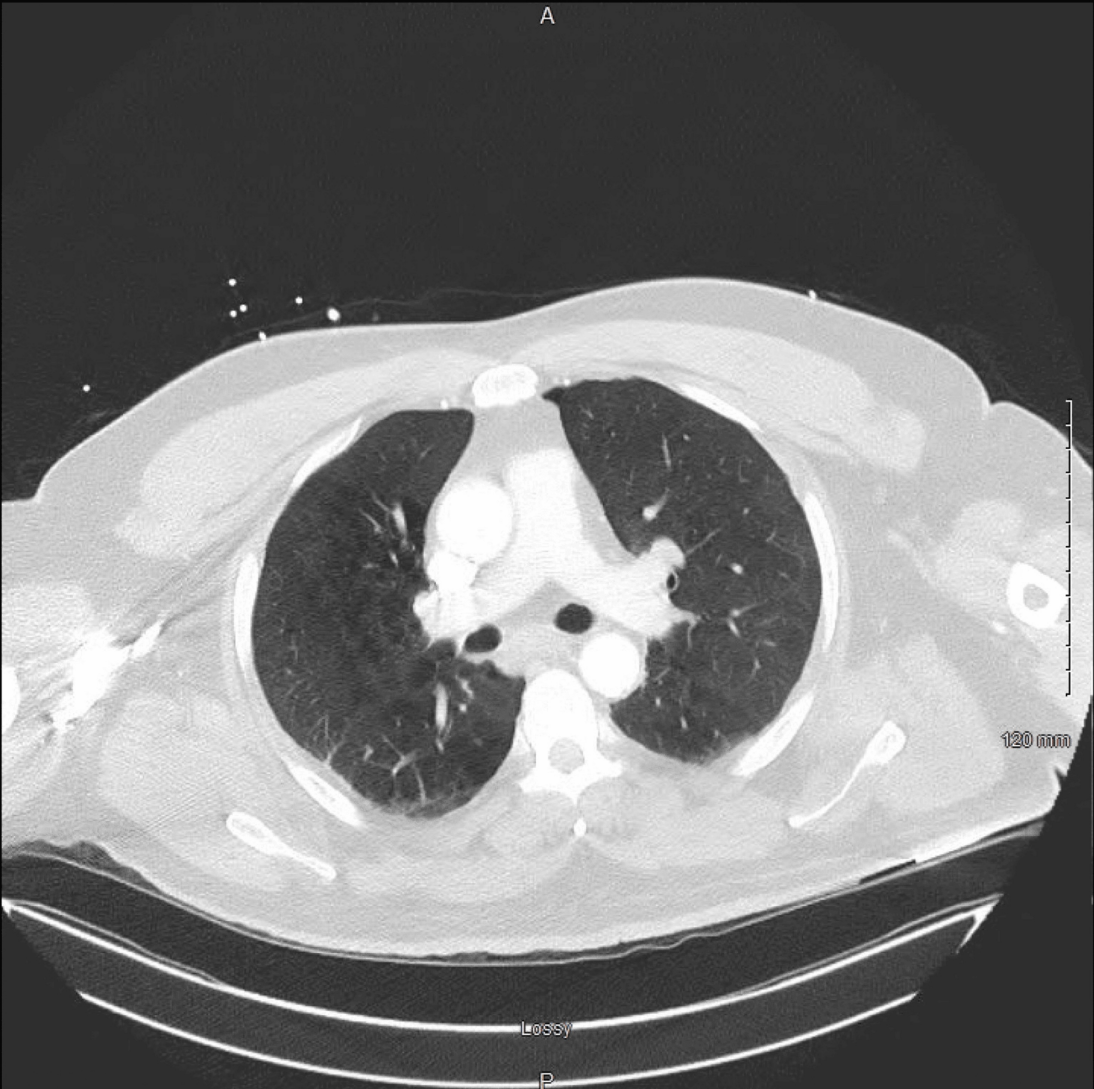
Chest CT scan A chest CT scan ordered initially to exclude the presence of pulmonary embolism. Notable in the picture are emphysematous changes

Considering the patient had a mild acute hypoxic respiratory failure with new-onset AF combined with the characteristic S1Q3T3 pattern on ECG and mildly elevated D-dimer, a CT angiography of the chest was urgently ordered to evaluate for PE, but results came back negative for PE. It was suspected that the patient had bilateral lung base atelectasis and diffuse emphysematous changes based on findings of the chest CT; however, it is notable that he mentioned he was a never-smoker. A-1 antitrypsin levels were also ordered, but levels came back normal (Figure 3).

**Figure 4:**
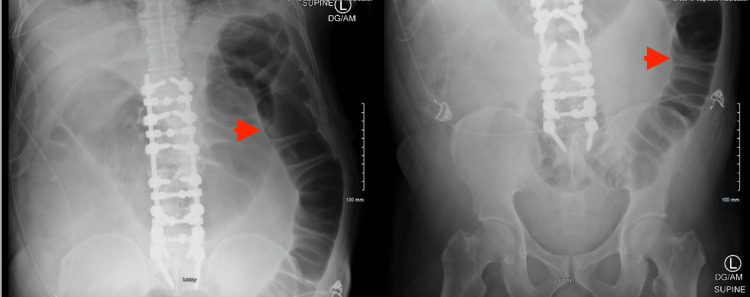
Abdominal X-ray Plain abdominal X-ray showing colonic dilation. It also showed the hardware of the spinal fusion surgery (red arrowheads)

**Figure 5:**
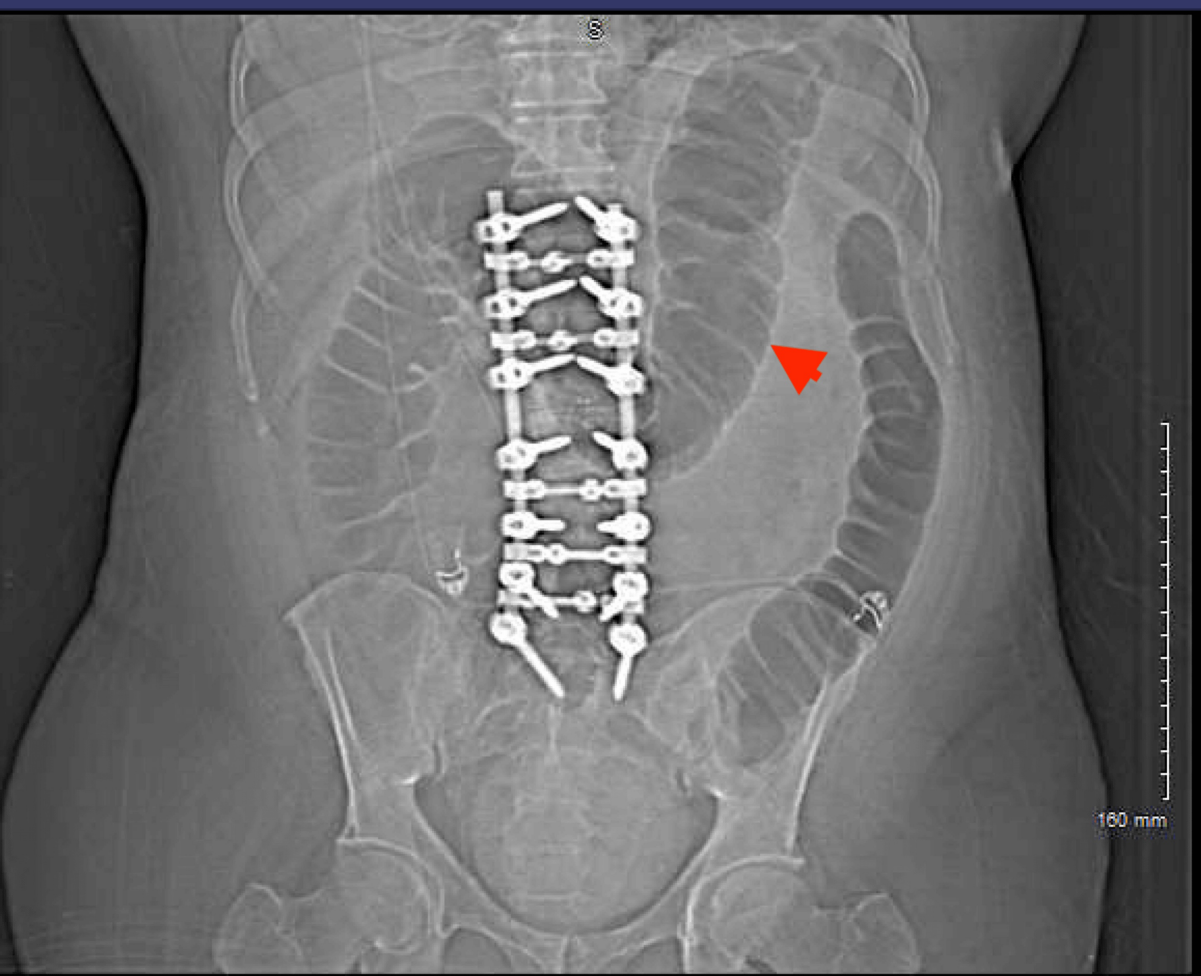
Abdominal CT scan A CT scan of the abdomen confirming the colonic dilation and ileus (red arrowheads)

**Figure 6:**
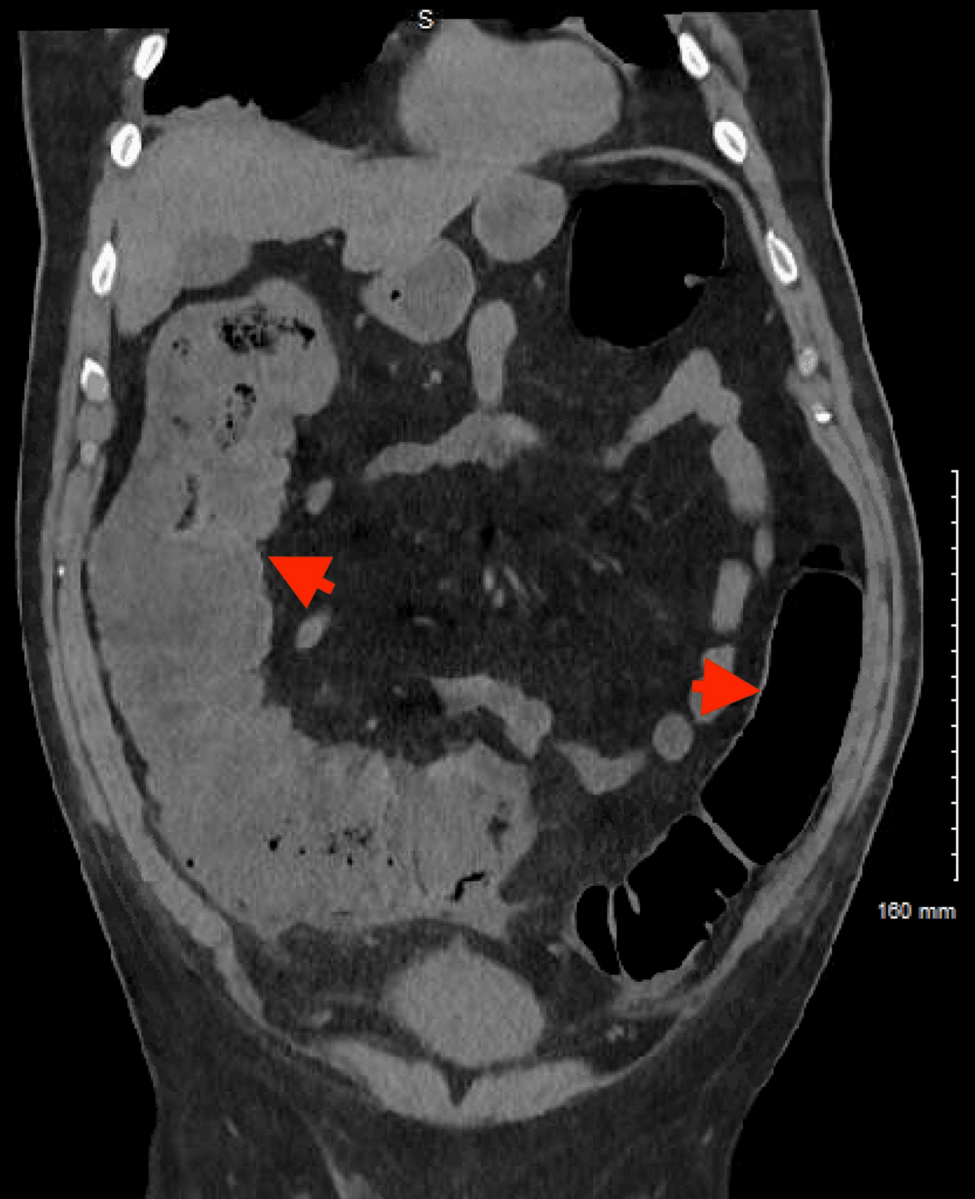
Abdominal CT scan CT scan of the abdomen confirming the colonic dilation and ileus (red arrowheads)

The CT did suggest colonic ileus with the transverse colon measuring approximately 70 mm at the largest diameter, which was confirmed by plain abdominal X-ray (Figure 4). Similar findings were confirmed with an abdominal CT scan as well, without transition points or masses (Figures 5, 6).

The patient was admitted to the medical floor and was started on oral metoprolol succinate 100 mg daily, diltiazem 30 mg every six hours (which was later discontinued upon discussion with the cardiology team), and apixaban 5 mg twice daily (started on day 8 postoperatively considering the need for anticoagulation in the setting of atrial fibrillation and a congestive heart failure, hypertension, age 75 years or older, diabetes, stroke or transient ischemic attack, vascular disease, age 65-74 years, sex category: female {CHA2DS2-VASc} score of 2). A transthoracic echocardiogram showed a small left ventricular cavity with hyperdynamic left ventricular systolic function, a left ventricular ejection fraction (LVEF) of 70%, normal left ventricular wall thickness, and grade I (mild) diastolic dysfunction. The right ventricle cavity was noted to be small with hyperdynamic right ventricular systolic function. No right ventricular pressures were charted in the report, and no valvular abnormalities were seen. For colonic ileus, he was treated with bowel rest, electrolyte replacement, IV fluids, pain control but minimizing opioids, and gradual introduction of feeding.

Over the next couple of days, he continued to require supplemental oxygen, and another D-dimer test was obtained and resulted in an elevation to 1095 ng/mL. Another ECG was obtained, which was still showing sinus tachycardia and the persistence of the S1Q3T3 pattern. Suspicion for PE remained high, thus another CT angiography of the chest was ordered and again failed to demonstrate the presence of PE.

**Figure 7:**
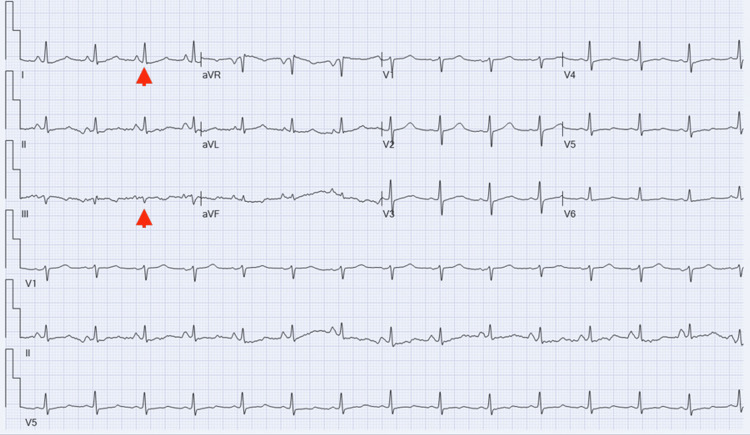
Final ECG ECG showing sinus rhythm with the resolution of the S1Q3T3 pattern (red arrowheads) ECG: electrocardiogram

In the subsequent days, the patient reported improvement in abdominal distention and the consistency of stool and was able to tolerate a regular diet. A follow-up ECG was ordered and revealed low-voltage limb leads with the resolution of the tachycardia and the S1Q3T3 pattern (Figure 7). The patient was safely discharged home with home health for physical therapy and expectations to have a follow-up appointment with a cardiologist on an outpatient basis.

## Discussion

Traditionally, the S1Q3T3 ECG pattern has commonly been associated with PE and right heart strain, but our findings add to the body of literature suggesting that this may not always be the case. Studies mention the S1Q3T3 pattern seen in patients with pneumothorax, acute asthma exacerbation during pregnancy, pneumonia, large pleural effusion, massive disseminated cryptococcal infection causing non-thrombotic pulmonary embolism, and IVC compression secondary to metastatic gastric adenocarcinoma and even in healthy patients [14-22]. Interestingly, all these entities can cause a direct or indirect RV strain and/or RV dilation, which is the proposed underlying pathophysiology of this pattern, but none of them are associated with bowel pathology such as in our case. In our patient with colonic dilation and ileus, there may have hypothetically been increased luminal pressure that impaired venous drainage to the right atrium, potentially leading to an RV strain and hence the S1Q3T3 pattern on ECG. Nevertheless, we do not have imaging evidence of this. Had the echocardiogram revealed low right atrial pressures or a small/collapsed IVC, this could have been projected to be the evidence of decreased venous return to the right ventricle as a result of increased intraabdominal pressure secondary to colonic ileus.

The case of gastric carcinoma compressing the IVC and leading to the S1Q3T3 pattern may also be related to impaired venous drainage leading to RV [19]. While we hypothesize that both cases are similar in pathogenesis due to the effect of IVC compression, with the presence of tachycardia in both instances possibly hinting toward decreased preload resulting from venous pooling distal to the IVC, it is also important to shed light on the differences including the absence of lower extremity edema in our colonic ileus case in addition to the acute nature of IVC compression in our case, which was likely due to postoperative complication after lumbar spinal fusion as opposed to the more chronic nature in the gastric adenocarcinoma case. In this case, colonic ileus responded well to conservative and supportive measures after a few days of hospitalization. Other factors could have played a role in the emergence of this pattern including tachycardia that was evident on physical examination, hypoxia, and bilateral lower lobe atelectasis that was seen on chest X-ray (CXR), which later improved after persistent physical therapy sessions and the encouragement of ambulation.

Given all the associations reported in the literature, the first differential diagnosis that is usually entertained by physicians in patients with the S1Q3T3 ECG pattern and acute presentation is PE, such as in our case. Most of the other causes that will come to mind are cardiopulmonary in origin, and rarely will physicians consider abdominal pathologies. It is possible that the S1Q3T3 pattern may have presented various etiologies beneath the diaphragm in real practice, but the association may not have been made and was overlooked. Once usual causes such as PE and pneumothorax were ruled out by CXR and CT imaging, we committed to searching for other possible causes leading to the discovery of the colonic ileus in this case. While not traditionally linked to ECG changes, we suspect that intraabdominal causes might have a role in the emergence of ECG changes at least through indirect mechanisms such as cases of IVC syndrome due to mass effect.

This highlights how this characteristic pattern can be very nonspecific to PE; thus, physicians should remember to approach this with broad differential diagnoses based on patients' presentations. It is imperative that we shed light on the connections discovered in what seemed to be unrelated components of the human body (ECG changes seen in non-cardiopulmonary etiologies) because it would allow us to collectively expand our knowledge and understanding. Recognizing this potential connection shall enable the practicing physician to change their perspective and break the cycle of fixating on certain disease processes, thus avoiding unnecessary repetition of nondiagnostic testing.

This case might also provide some guidance in the application of cardiac risk stratification prior to surgical intervention should this pattern be recognized during preoperative assessment. Investigations of the underlying causes for this pattern may provide evidence of chronic or, in similar cases, acute heart strain that could play a role in decision-making regarding the timing and the importance of said surgical intervention.

## Conclusions

The McGinn-White sign or the S1Q3T3 pattern is a very distinct ECG change, most commonly reported to be associated with acute PE, but many other associations have been found in the literature, some of which might not have a clear direct association, such as colonic dilation and ileus in our case. Similar cases may potentially allow physicians to expand their differential diagnoses beyond etiologies related to cardiac and pulmonary diseases and start considering abdominal pathologies as well, prompting an additional area of focus to physical examination and diagnostic testing. We aspire that this provides precedence against repetitive testing directed toward certain cardiopulmonary conditions, which will be in favor of mindful clinical practices aimed at reducing healthcare-related costs and avoiding hazards of radiographic imaging such as radiation exposure and contrast administration, ultimately leading to better overall patient-centered care.

It is imperative however to consider the limitations of the application of this hypothesized mechanism in clinical practice, as there has not yet been solid evidence reported on the emergence of the S1Q3T3 pattern in intraabdominal pathology. The application of this rationale to clinical use should be taken into the context of the patient as a whole and on a case-by-case basis as a possible indicator of an intraabdominal disease process should other, more common, causes be excluded first.
